# Resistant Hypertension in Nondialysis Chronic Kidney Disease

**DOI:** 10.1155/2013/929183

**Published:** 2013-04-22

**Authors:** Silvio Borrelli, Luca De Nicola, Giovanna Stanzione, Giuseppe Conte, Roberto Minutolo

**Affiliations:** Division of Nephrology, Second University of Naples, 80125 Naples, Italy

## Abstract

Resistant hypertension (RH) is defined as blood pressure (BP) that remains above the target of less than 140/90 mmHg in the general population and 130/80 mmHg in people with diabetes mellitus or chronic kidney disease (CKD) in spite of the use of at least three full-dose antihypertensive drugs including a diuretic or as BP that reaches the target by means of four or more drugs. In CKD, RH is a common condition due to a combination of factors including sodium retention, increased activity of the renin-angiotensin system, and enhanced activity of the sympathetic nervous system. Before defining the hypertensive patient as resistant it is mandatory to exclude the so-called “pseudoresistance.” This condition, which refers to the apparent failure to reach BP target in spite of an appropriate antihypertensive treatment, is mainly caused by white coat hypertension that is prevalent (30%) in CKD patients. Recently we have demonstrated that “true” RH represents an independent risk factor for renal and cardiovascular outcomes in CKD patients.

## 1. Definition and Prevalence of Resistant Hypertension in General Population

Hypertension is defined “resistant” (RH) when blood pressure (BP) levels persist above the therapeutic target (<140/90 mmHg for general population and <130/80 mmHg for patients with diabetes mellitus or chronic kidney disease (CKD)), despite the use of at least three antihypertensive drugs at full dose, including the diuretic. Furthermore, according to the current definition, also hypertensive patients who reach BP target by means of four or more drugs are considered resistant [1, 2].

Although the exact prevalence is unknown, several observational studies suggest that RH is a common clinical problem in general population [3–8]. In a recent analysis of NHANES 2003–2008, about 9% of 5,230 hypertensive patients can be identified as resistant to treatment. This prevalence increased to 13% when only treated patients were considered [3]. 

Main causes of RH are reported in Table 1. RH may be caused by biological-behavioral factors (such as smoking and obesity), drugs (NSAOIDs, steroids, and cyclosporine) or exogenous substances (liquirice, ginseng, etc.), and secondary causes of hypertension. Among these, CKD is most relevant for its epidemiological impact [8]. Indeed, the prevalence of CKD is rapidly rising worldwide with approximately 10% of the adult population currently affected [9]. Notably, 65–95% of CKD patients develop hypertension, as the glomerular filtration rate (GFR) declines from 85 to 15 mL/min [10], and hypertension is a determinant of progression of renal damage, especially in proteinuric and diabetic patients [11, 12], and of cardiovascular risk [13].

## 2. Pseudoresistance

Before defining the hypertensive patient as resistant it is mandatory to exclude the so-called “pseudoresistance.” This condition refers to the “apparent” failure to reach BP target, in spite of an appropriate antihypertensive treatment. Among the causes of pseudoresistance (Table 2), the most frequent is represented by the presence of white coat hypertension (WCH). Ambulatory blood pressure monitoring (ABPM) or home blood pressure (HBP) allows the identification of white coat effect defined by the coexistence of persistently high office BP with normal ABP or HBP. Therefore, out-of-office monitoring of BP is the essential tool for correctly diagnosing RH. Indeed, in the Spanish ABP registry, 12% of the 68,045 patients examined were diagnosed as RH; however, after ABP monitoring, as many as 37% of them were identified as pseudoresistant [14].

The second critical aspect for excluding pseudoresistance is the assessment of adherence to antihypertensive therapy. Lack of adherence is frequently encountered in clinical practice; indeed, nearly half of patients with hypertension withdraw therapy within the first year after diagnosis [15], and that, over 10 years of follow-up, about 40% of patients discontinue permanently antihypertensive drugs [15, 16]. The main causes of poor compliance are represented mainly by the fear of side effects, complicated treatment plans, poor doctor-patient communication, and costs of therapy (Table 2).

## 3. Resistant Hypertension in CKD Patients

**Figure 1:**
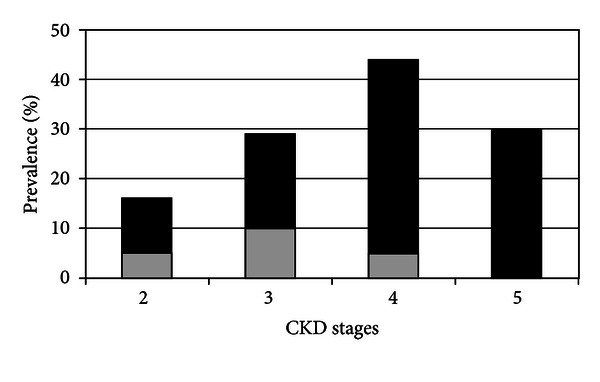
Prevalence of true resistance (black bar) and pseudoresistance (gray bar) in CKD stages [17].

CKD is at the same time cause and complication of poorly controlled hypertension. The evaluation of RH in CKD patients is highly relevant for two main reasons. First, RH is common in CKD patients, and its prevalence increases with worsening of kidney damage (Figure 1) [17]. Second, RH represents an independent risk factor for renal, and cardiovascular (CV) outcomes in CKD patients [17, 18].

Several surveys in CKD patients have demonstrated a high incidence of uncontrolled hypertension in clinical practice. Indeed the BP target is reached in only a small proportion (10–20%), both in nephrology and nonnephrology settings [19–24]. 

**Figure 2:**
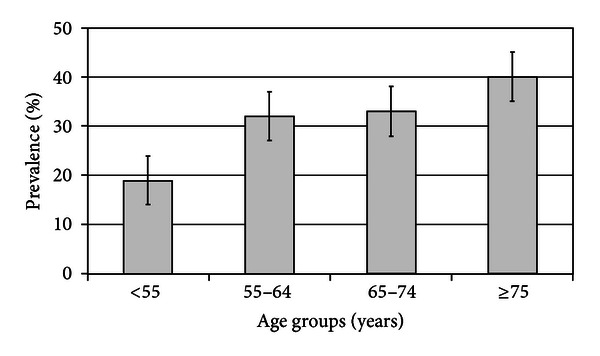
Prevalence of white coat hypertension in CKD patients stratified by age [26]. *P* = 0.001 for trend.

However, uncontrolled hypertension is not equivalent of RH. This is particularly true in CKD patients in whom WCH is a common feature. Indeed, we have previously reported that WCH occurred in about 30% of patient with office BP ≥ 130/80 mmHg [25], and, as illustrated in Figure 2, its prevalence increased with aging [26]. More recently, our group has confirmed this finding in a larger cohort of CKD patients evidencing that pseudoresistance involved 24% of patients [17], defined as resistant only on the basis of office BP and drug number [1]. Of note, prevalence of pseudoresistance is typically encountered in early stages of CKD and virtually disappeared in CKD stage 5 (Figure 1) [17].

Contemporaneous assessment of ABP monitoring, therefore, allowed to disclose a prevalence of “true” RH of 23–25% in CKD patients [17, 18], that corresponds to a prevalence three times greater than that reported in essential hypertension (~8%) [14]. In addition, when nephrologists intensify antihypertensive therapy to reach BP target, the prevalence of RH increases from 26% to 38%. Our retrospective study also evidenced that diabetes and proteinuria are main determinants of RH [18].

## 4. Pathogenesis of RH in CKD Patients

**Figure 3:**
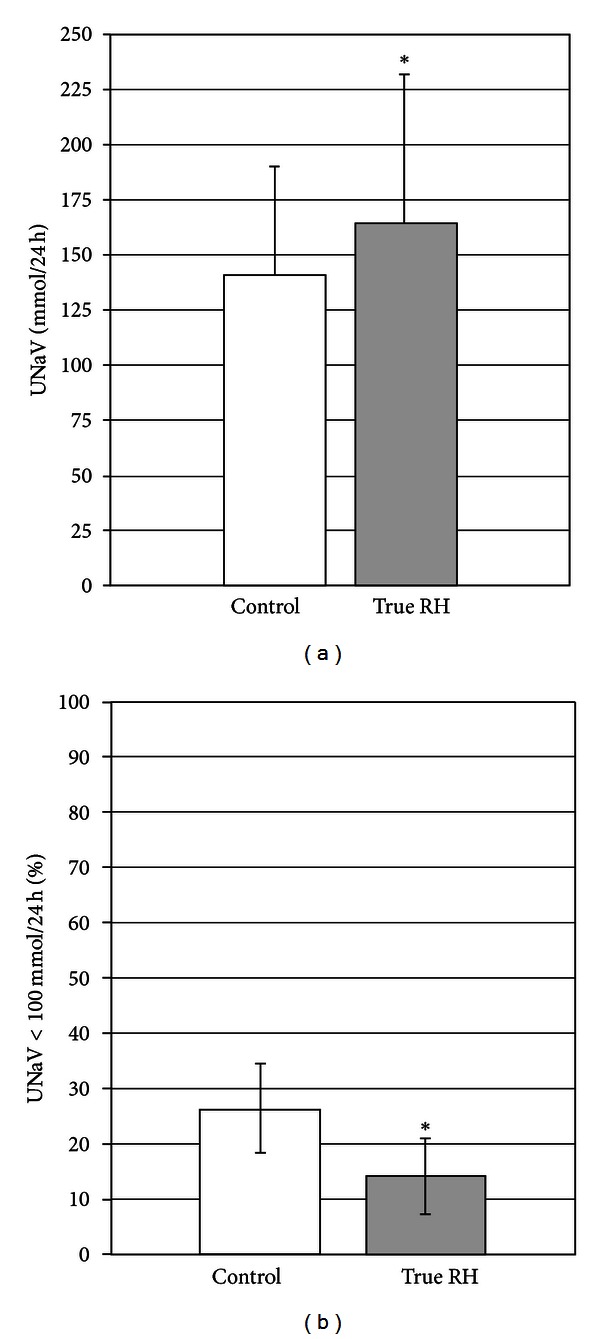
Mean urinary sodium excretion (UnaV, mmol/24 h) and prevalence of low-salt diet (UnaV < 100 mmol/die, %) in CKD patients with controlled BP (control, white bars) and with true RH (gray, bars) [17]. *P < 0.05 versus controls.

The pathogenesis of hypertension in CKD is multifactorial being a combination of factors including sodium retention, increased activity of the renin-angiotensin system, and enhanced activity of the sympathetic nervous system; this may in part justify the low success rate of antihypertensive treatment [27]. The most frequent pathophysiological disorder is the salt and water retention occurring in the majority of patients with reduced glomerular filtration rate (GFR). The resulting expansion of the ECV allows preserving the external balance of sodium, but with consequent development of persistent and often refractory hypertension therapy. In these patients, the entity of ECV expansion is directly dependent on the degree of GFR impairment and corresponds to approximately 5% to 10% of body weight, even in the absence of peripheral edema [28]. Of note, the salt sensitivity of BP is not a feature limited to the advanced stages of renal disease, but begins before the development of clear hypertension and severe GFR decline [29, 30]. The fact that sodium excretion is commonly impaired in CKD is further testified by the large prevalence of nocturnal hypertension in CKD as compared to essential hypertension [31–33]. Furthermore, in CKD patients, systemic hypertension is sustained by the activation of renin-angiotensin system (RAS), which is inappropriate compared to expansion of the ECV. The ensuing glomerular hyperfiltration leads to the progressive kidney damage in the long term. The institution of measures to help prevent this process, such as antihypertensive therapy with an angiotensin converting enzyme inhibitor or an angiotensin II receptor blocker, may slow progressive disease and even preserve renal function [34]. It is of interest that in our prospective study [17], we found that mean urinary excretion of sodium was higher in RH patients (*P* = 0.004), and consequently the adherence to low-salt diet was poorer (*P* = 0.026) (Figure 3).

Therapeutic interventions aimed to inhibit the SRA and to reduce the ECV expansion frequently are insufficient at normalizing hypertension status in these patients, suggesting additional mechanisms in the pathophysiology of CKD-related hypertension. A series of experimental observations has allowed, in fact, generating new hypotheses such as the increase adrenergic activity, secondary hyperparathyroidism, and dysregulation of endothelial factors regulating the contractility of smooth muscle vessel [27]. Finally, some lines of evidence have recently indicated that sleep disturbances prevalence is higher in subjects with CKD. Given that sleep apnea might be a cause of RH (Table 1), it might contribute to explain the high prevalence of RH in subjects with CKD [35].

## 5. Prognosis of RH in CKD 

In the population with essential hypertension, a relationship between RH and cardiovascular risk has been reported [36, 37]. Furthermore, studies have shown that presence of mild-to-moderate GFR reduction and/or microalbuminuria amplifies the cardiovascular risk correlated to RH in the general hypertensive population [38, 39]. However, only two studies evaluated the prognostic role of RH in patients with established CKD and more advanced renal damage [17, 18]. In a retrospective study, we evidenced that RH was associated with greater risk of renal death (HR 1.85, 95% CI, 1.13–3.03), independently from main clinical features and degree of BP control [18]. This finding has been confirmed by our group in a very recent prospective study in a cohort of 436 hypertensive CKD patients under nephrology care [17]. In that study, we assessed the risk of ESRD and fatal and nonfatal CV events in CKD patients stratified by presence of hypertension with and without RH. During 52.0 months of follow-up, 165 renal events and 109 fatal and non-fatal CV events were documented. Patients with normal ABP had the best prognosis for either outcome independent of their RH status, whereas the highest risk for cardio-renal events was observed only in true resistance. Indeed, in comparison with sustained hypertension, true resistance predicted CV risk (HR 2.05, 95% CI, 1.23–3.43) but not renal risk (HR 1.23, 95% CI, 0.83–1.82). 

Of note, in pseudoresistant patients, ABP profiles, target organ damage (prevalence of LVH and severity of renal disease) did not differ from normotensive patients, and their cardio-renal outcome was comparable to that of control patients [17]. This result is clinically relevant and supports the need to identify pseudoresistant CKD patients to avoid aggressive antihypertensive therapy. Indeed, these patients were characterized by systolic BP levels during daytime, and especially at nighttime, close to the threshold limit of hypoperfusion (100 mmHg). Under these circumstances, a tighter control of BP merely based on the detection of elevated BP in office may expose patients to ischemia-induced worsening of cardio-renal damage [40, 41] and eventually convert their prognosis from favourable to unfavourable.

The mechanisms underlying the different prognostic value of RH are not readily apparent; however, we can hypothesize that persistence of hypertension despite optimal antihypertensive treatment specifically identifies patients with more severe vascular damage. Diabetes, left ventricular hypertrophy, higher proteinuria, and, as mentioned, high salt intake, variables that we found independently associated with true resistance, are in fact all associated with endothelial dysfunction and arterial stiffness [42–45]. In particular, CKD has shown that proteinuria, rather than GFR, relates to the severity of hypertension [46]. Indeed, although low GFR is recognized as a CV risk factor [47], proteinuria in CKD patients is considered a better marker of the presence of vascular disease [48, 49].

## 6. Possible Therapeutic Interventions in RH Patients with CKD

The multifactorial pathogenesis of hypertension in CKD imposes a multilevel treatment in these patients, even though to date there are no studies that assessed whether a particular combination of antihypertensive drugs is most advantageous to control hypertension in RH patients. The AASK study suggested that use of dihydropyridinic calcium-channel blocker (amlodipine) might not be adequate for use in CKD subjects, possibly because of increasing the GFR and thus inducing glomerular damage [50]. More recently, REIN-2 study has shown that felodipine (dihydropyridinic calcium-channel blocker) added to a background therapy with CEI may be safely used in CKD individuals for reaching low BP target, but it did not reduce the progression to ESRD [51]. Finally, a recent systematic review suggested that the treatment with beta-blockers improved all-cause mortality in patients with CKD and heart failure [52], but these drugs did not appear having a renoprotective effect [50].

In CKD patients with RH, the pivotal intervention is certainly represented by the restriction of sodium intake. This dietary measure, however, is scarcely implemented as testified by the poor adherence (~20%) to low sodium diet even in patients regularly followed in nephrology clinics [53]. This is a paradoxical condition if one considers the high salt sensitivity of CKD as well as the positive results obtained in the few pilot studies published to date. Koomans et al. found that lower sodium intake markedly diminished BP in patients with advanced CKD [54]. Interestingly, we suggested that salt restriction may contribute to the improvement in renal outcome observed in CKD patients treated with or without dietary protein restriction [29, 55]. Finally, it also is well recognized that a reduction in daily sodium intake enhances the antihypertensive and antiproteinuric effects of converting enzyme inhibitors [56]. More importantly, a small randomized cross-over trial of dietary salt restriction in RH patients has demonstrated that low-salt diet significantly decreased office systolic and diastolic BP (by 23 and 9 mmHg, resp.) and 24-h BP from 150/82 to 130/72 mmHg [57]. 

The critical role of salt retention in CKD-dependent hypertension precludes optimal control of BP during pharmacological treatment with antihypertensive agents, especially vasodilators [28] that are prescribed in almost half of CKD patients [53]. Indeed, early studies have shown that, to obtain full expression of the antihypertensive effects of minoxidil, a potent vasodilating agent, it is necessary to antagonize its antinatriuretic side effects by coadministering a diuretic agent or limiting the vasodilation-induced activation of the renin-angiotensin and adrenergic nervous systems [58, 59].

Improving compliance to a low-salt diet in hypertensive patients with CKD is helpful to make patient aware of the importance of low-salt intake to prevent progression of CV disease and CKD, to instruct patient on the correct way to collect 24-hour urine specimen, to monitor 24-hour urinary sodium excretion at each visit (target, 100 mmol/day), to communicate to patient the daily salt intake estimated by 24-hour urinary sodium excretion (target, 6 g/day), and to give specific recommendations on sodium restriction in diet listed in the following list.

 Ten recommendations to restrict sodium in your diet:
(1) move the salt shaker away from the table;
(2) cook pasta, rice, and cereals without salt (add in smaller amount directly on cooked food);
(3) in cooking and at the table, increase the use of spices (e.g., herbs, lemon, vinegar, and hot pepper);
(4) abolish salt-containing condiments (e.g., ketchup, mayonnaise, mustard, and barbecue sauce);
(5) look for the amount of sodium on food labels;
(6) look for low-salt bread and fresh or plain frozen foods;
(7) cut down frozen dinners, canned soups, packaged mixes, cured meat and fish (e.g., ham, bacon, anchovies, and salmon);
(8) choose fresh rather than seasoned cheese;
(9) rinse canned foods (e.g., tuna) to remove some sodium;
(10) abolish salty snack foods (e.g., chips, nuts).

Generally diuretics are included in the definition of RH; however, in CKD patients the most crucial task of diuretic therapy is to properly select class and dose in relation to the level of kidney function. Indeed, if patients with mild renal impairment (GFR > 40 mL/min/1.73 m^2^) may respond to thiazide diuretics, those with more advanced CKD require the use of more potent loop diuretics administered at doses proportional to the reduced GFR [60]. In a clinical trial performed in patients with GFR in the range 10–40 mL/min, correction of volume expansion, as evidenced by a decrement in body weight of approximately 2.0 kg and a parallel marked reduction in BP, was safely induced by oral administration of only furosemide at doses inversely proportional to GFR level (1.0, 2.5, and 4.0 mg/kg body weight per day in patients with GFRs of 40–31, 30–20, and 19–10 mL/min, resp.) [61]. Therefore to improve the modalities of diuretic treatment is helpful to begin with a low dose and gradually increase the dose to obtain progressive reduction of the body weight (0.5–1 kg/day) until to correction of sodium retention. Alternatively, the diuretic resistance can be overcome with the addition of thiazides, such as metolazone, that blocks the reabsorption of sodium in the distal segments, thereby reducing the breaking phenomenon [62]. Disappointingly enough, nephrologists are today still reluctant to adequately use loop diuretics in their hypertensive CKD patients. This erroneous attitude cannot be justified by the fear of side effects, which are infrequent, usually reversible and predictable when the patient is checked periodically [53, 63]. 

A further diuretic agent successfully tested in RH patients is spironolactone based on the finding that plasma aldosterone levels are higher in RH that in those with controlled hypertension [64]. Recently, ASPIRANT study, a randomized, controlled, double-blind study evaluated the antihypertensive effects of spironolactone in 117 patients with RH. Spironolactone was administered at doses of 25 mg/day for 8 weeks in addition to the preexisting therapy. At the end of 8 weeks of the study, systolic BP (both measured in the office and outpatient) was significantly reduced in treated patients in the absence of adverse effects [65]. However, despite their efficacy, antialdosterone drugs must be used very carefully in CKD with advanced disease (GFR < 30 mL/min/1.73 m^2^) due to the higher risk of hyperkalemia.

A novel therapeutic approach for RH is represented by catheter-based radiofrequency ablation of the renal sympathetic nerves, which was originally proposed in essential hypertension [1, 2]. More recently, one study assessed this intervention in moderate-to-severe CKD [66]. Fifteen patients with GFR < 45 mL/min/1.73 m^2^ were successfully treated with renal ablation with a significant systolic/diastolic BP reduction being evident after 1 month from intervention (−34/−12 mmHg) and persisting at one year (−33/−19 mmHg) [66]. However, this is a small study with relatively short-term follow-up, and ultimate safety and efficacy of the catheter-based renal denervation procedure must await longer follow-up in a larger group of patients with CKD; indeed, of the five patients followed for 12 months, eGFR appears to have declined precipitously in one and more gradually in three others compared with the value at 6 months [66]. A trial in a larger group of patients is now underway prior to seeking approval from the Food and Drug Administration for approval of the radio catheter device. However, we are at the very beginning of the use of this invasive approach, and more data are needed before claiming for a therapeutic success. This holds true for carotid baroreceptor stimulator that has been tested in essential resistant hypertension [67] but not in CKD patients.

## 7. Conclusions

RH is a common condition in CKD due to a combination of factors including sodium retention, increased activity of the renin-angiotensin system, and enhanced activity of the sympathetic nervous system. However, the higher prevalence of WCH in these patients imposes an out-of-office monitoring (ABPM or HBP) to distinguish between pseudoresistance and true RH. Therefore a more large use of ABPM in CKD patients is auspicable to attempt to limit the misclassification of hypertensive status in order to avoid unnecessary aggressive antihypertensive medication. To date the degree to which cardiovascular risk is reduced with treatment of resistant hypertension is unknown. Catheter-based radiofrequency ablation of the renal sympathetic nerves has been proposed, even though a greater implementation of a low-salt diet and a adequate use of the diuretic may be the first-choice therapeutic approach for controlling RH in CKD patients.

## Figures and Tables

**Table 1:** Determinants of resistant hypertension in general population.

Clinical condition	
Diabetes mellitus	
Older age	
Obesity	
Drugs	
Nonsteroidal anti-inflammatory drugs	
Corticosteroids	
Oral contraceptive hormones	
Erythropoietin	
Cyclosporine and tacrolimus	
Sympathomimetics (decongestants)	
Exogenous substances	
Tobacco	
Alcohol	
Cocaine, amphetamines, and other illicit drugs	
Licorice	
Herbal supplements (ginseng, yohimbine)	
Secondary causes	
Common	
Chronic Kidney disease	
Primary aldosteronism	
Sleep apnea	
Hyper-hypothyroidism	
Renal artery disease	
Uncommon	
Cushing's syndrome	
Pheochromocytoma	
Aortic coarctation	
Hyperparathyroidism	

**Table 2:** Causes of pseudoresistance.

White coat effect	
Adherence therapy	
Side effect of medication	
Complicated dosing schedules	
Poor relation between doctor and patients	
Costs of medication	
Improper blood pressure measurement	
Incorrect cuff size	
Related to antihypertensive medication	
Inadequate doses of diuretic	
Inappropriate combination	
